# Emerging Trends in Bioinspired Superhydrophobic and Superoleophobic Sustainable Surfaces

**DOI:** 10.1002/adma.202415961

**Published:** 2025-02-18

**Authors:** Cerys M. Cormican, Sinem Bektaş, Francisco J. Martin‐Martinez, Shirin Alexander

**Affiliations:** ^1^ Faculty of Science and Engineering Department of Chemical Engineering Swansea University Bay Campus Fabian Way Swansea SA1 8EN UK; ^2^ Faculty of Science and Engineering Department of Materials Science and Engineering Swansea University Bay Campus Fabian Way Swansea SA1 8EN UK; ^3^ Faculty of Natural Mathematical and Engineering Sciences Department of Chemistry King's College London London SE1 1DB UK

**Keywords:** bioinspired materials, biomimetics, superhydrophobicity, superoleophobicity, sustainability

## Abstract

Inspired by nature's ability to master materials for performance and sustainability, biomimicry has enabled the creation of bioinspired materials for structural color, superadhesion, hydrophobicity and hydrophilicity, among many others. This review summarizes the emerging trends in novel sustainable fluorocarbon‐free bioinspired designs for creating superhydrophobic and superoleophobic surfaces. It discusses methods, challenges, and future directions, alongside the impact of computational modeling and artificial intelligence in accelerating the experimental development of more sustainable surface materials. While significant progress is made in superhydrophobic materials, sustainable superoleophobic surfaces remain a challenge. However, bioinspiration and experimental techniques supported by computational platforms are paving the way to new renewable and biodegradable repellent surfaces that meet environmental standards without sacrificing performance. Nevertheless, despite environmental concerns, and policies, several bioinspired designs still continue to apply fluorination and other environmentally harmful techniques to achieve the required standard of repellency. As discussed in this critical review, a new paradigm that integrates advanced materials characterization, nanotechnology, additive manufacturing, computational modeling, and artificial intelligence is coming, to generate bioinspired materials with tailored superhydrophobicity and superoleophobicity while adhering to environmental standards.

## Graphical abstract

Biomimicry offers an exceptional opportunity to design materials with advanced properties. This review summarizes the latest findings for developing sustainable superhydrophobic–superoleophobic materials using biomimicry, their challenges, and future directions. For a more comprehensive approach in this research area, experimental methods for surface engineering are given in detail, along with computational modeling and artificial intelligence applications.

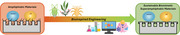

## 1 Introduction

Nature has more than 3.8 billion years of experience in material design at the pinnacle of performance and sustainable technologies. Only the most effective designs and efficient procedures have managed to propagate and stand the test of time. Since antiquity, humanity has taken inspiration from biology to advance contemporary technologies. Tools and weapons, clothing, and endless examples of aircrafts, helicopters, drones, and even wind turbines or urban planning have been developed with biomimicry.^[^ ^1^, ^2^, ^3^, ^4^, ^5^ ^]^

Nanostructured surfaces have been very specific sources of inspiration. Geckos possess spatula surface nanostructure on their feet, which maximizes surface contact and van der Waals forces to adhere to walls. This has resulted in the development of Gecko‐inspired coatings and adhesives used in a range of fields including medical and electronic.^[^ ^5^ ^]^ Velcro was invented after a Swiss engineer noticed burrs sticking to his dog's fur due to their microscale hooks clinging to the substrate.^[^ ^6^ ^]^ Even fireflies known for their ability to emit light benefit from high‐transmission surface nanostructures that enhance light output. This resulted in firefly‐inspired LEDs with improved illumination and reduced energy consumption, as well as a longer lifespan than traditional bulbs.^[^ ^7^ ^]^ Natural photonic crystals and structural color have provided inspiration for a huge range of applications such as displays, sensors, optics, medical fields, solar cells, camouflage, and paints.^[^ ^8^, ^9^ ^]^ The Kingfisher's splash‐minimizing dive is a result of the aerodynamics of the beak design, which was mimicked by engineers to develop the bullet train, reducing the noise pollution and pressure waves, as well as improving the speed and energy efficiency.^[^ ^10^ ^]^ Undoubtedly, nature has a myriad of innovations, developed to promote survival and success in withstanding the dynamics of the perpetually changing environment, which hold elite solutions to aid in the design of advanced materials.

Fascinated by these Natural designs, modern technology, with both computational and experimental techniques, has allowed humanity to mimic biota. There have been effective recent reviews regarding experimental techniques to produce superhydrophobic materials, superoleophobic materials, and bioinspired superhydrophobic materials. In this review we combine superhydrophobicity, superoleophobicity, bioinspiration, and sustainability, to provide an up to date and critical examination of the research field and current techniques encompassing all aspects from performance to environmental effects using both experimental and computational approaches. Nowadays, there are tools to observe, characterize, and recreate some natural designs from the nanoscale to the macroscale. Some examples include electrospinning, 3D printing, molding, hot embossing, lasering, lithography, oxygen plasma treatments, pyrolysis, sol–gel process, magnetron sputtering deposition, and even the use of nanomaterials or photonic crystals. Electrospinning increases the surface roughness to enhance the wetting properties of the material.^[^ ^11^, ^12^, ^13^ ^]^ 3D printing creates the necessary topography required for superhydrophobic/oleophobic materials, although it is limited to the microscale, preventing the creation of nanostructured designs as intricate as those in nature.^[^ ^14^, ^15^, ^16^, ^17^ ^]^ Molding and templating have been used to create biomimetic structures with re‐entrant topography capable of repelling low‐surface‐tension liquids.^[^ ^18^ ^]^ Similarly, hot embossing is able to create micro‐ and nanostructures on polymeric surfaces with superhydrophobic properties.^[^ ^19^ ^]^ Lasering creates surface textures on metals, nonmetals, and composite materials.^[^ ^20^, ^21^ ^]^ Lithography and etching provide highly geometrically controllable surfaces of organized arrays through various size scales, tuneable for desired properties.^[^ ^22^ ^]^ Oxygen plasma treatment oxidizes the surface to create hydrophilic polar groups.^[^ ^23^, ^24^ ^]^ Pyrolysis increases liquid repellency of a material, while sol–gel processes create xerogels and aerogels with hydrophobic/oleophobic properties.^[^ ^25^, ^26^, ^27^, ^28^, ^29^, ^30^ ^]^ Magnetron sputtering deposition can also provide hydrophobic and oleophobic thin films, although they are often lacking stability and durability.^[^ ^31^ ^]^

Nanomaterials are also utilized to increase the surface roughness and to enhance the wettability and surface area to volume ratio of material surfaces, with different properties from the corresponding bulk material.^[^ ^32^, ^33^, ^34^, ^35^ ^]^ Thus, the use of superhydrophobic hybrid nanocomposites is an emerging field with potential to overcome stability and durability issues by directing the nanostructures downward creating nanopores or similar.^[^ ^36^ ^]^ Also, photonic crystals have complex surface geometries and chemical compositions which can be tuned toward the desired wettability properties.^[^ ^37^ ^]^ Layer‐by‐layer (self‐)assembled materials and self‐assembled monolayers (SAMs) can also display hydro/oleophobicity.^[^ ^38^, ^39^, ^40^ ^]^

Additionally, developments in computational hardware and software alongside advancements in machine learning and artificial intelligence is accelerating the design of superhydrophobic and superoleophobic materials. Thus, a new paradigm that integrates advanced materials characterization, nanotechnology, additive manufacturing and computational modeling and artificial intelligence is coming to generate bioinspired materials for optimal performance, while adhering to environmental standards.

### 1.1 Hydrophobicity in Nature

**Figure 1:**
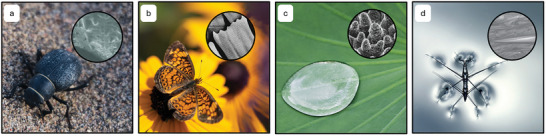
Hydrophobicity in nature. a) A Namib Beetle (*Stenocara gracilipes*) and an SEM image of its surface features. Reproduced with permission.^[^ ^44^ ^]^ Copyright 2010, Nørgaard and Dacke. b) A butterfly and an SEM image of the primary rectangular scale structure of its wing. Reproduced with permission.^[^ ^51^ ^]^ Copyright 2014, Science China Press. c) A lotus leaf and an SEM image of its microstructure. Reproduced with permission.^[^ ^60^ ^]^ Copyright 2011, Beilstein‐Institut. d) A water strider and an optical microscope image of its leg, showing the bristles, setae, and microtrichia microstructures. Reproduced with permission.^[^ ^66^ ^]^ Copyright 2004, Springer Nature Limited.

Many intricate designs in the biosphere have been perfectly fine‐tuned for superhydrophobicity. For example, the fogstand beetles (*Onymacris unguicularis*) and other Namib beetles (*O. laeviceps*, *Stenocara gracilipes*, *Physasterna cribripes*), which reside in the Namib Desert have very little access to water.^[^ ^41^, ^42^, ^43^, ^44^ ^]^ To combat this, they have developed a unique ability to obtain water from humid air due to the chemistry and structure of their backs (**Figure** **1**a).^[^ ^42^, ^45^ ^]^ They have an abundance of microscale bumps/ridges on the surface of their backs, which are uncoated and hydrophilic.^[^ ^45^, ^46^ ^]^ The areas which are not bumps are wax coated and therefore hydrophobic. This means water from fog can collect on the hydrophilic peaks, and drip down to the hydrophobic zone, where in combination with the head down stance of the beetle, the water can roll freely over the hydrophobic surface toward the beetle's mouth where it can drink the collected water. This has been a notable source of interest for synthetic fog harvesting opportunities.^[^ ^47^, ^48^ ^]^ Another example of hydrophobicity in nature is butterfly wings, showing self‐cleaning and anti‐icing properties (Figure 1b).^[^ ^49^ ^]^ Many butterfly species have chitin‐based wings composed of periodic microsized rectangular scales adorned with a secondary structure of nanoscale longitudinal ridges and lateral bridges as well as nanostrips atop the secondary structure, resulting in a three‐phase hierarchical micro/nanostructure.^[^ ^50^, ^51^ ^]^ As a result of this surface structure, butterflies have been identified to have high water contact angles (WCAs), sometimes even in the superhydrophobic region.^[^ ^50^, ^52^ ^]^ An abundance of plant species such as rose petals and rice leaves exhibit (super)hydrophobic surfaces due to either surface structures, cuticle waxes and coatings, or a combination of both.^[^ ^53^, ^54^ ^]^ Water‐repellent leaves possess a wax layer containing crystals from 0.5 to 20 µm in length and varying from large observable wax crystals clusters to small consistent crystals appearing as a smooth uniform layer.^[^ ^55^ ^]^ Furthermore, leaves with wax covered trichromes are seen to be exceptionally hydrophobic.^[^ ^55^, ^56^ ^]^ Depending on the species of the plant, leaves can have considerably varying surface structures as well as wax types. Overall, the most consistently hydrophobic leaf types are those with low carbonyl species present, ordered platelet light structures with a high degree of roughness and high polar and Lewis base free energies.^[^ ^57^ ^]^ The most prominent example is the lotus leaf (*Nelumbo nucifera*). Lotus leaves have WCAs over 150° and water sliding angles (WSAs) <5° endowing them with supreme superhydrophobic and self‐cleaning properties, leading to widespread recognition of the term the lotus effect to describe self‐cleaning surfaces (Figure 1c).^[^ ^55^, ^58^, ^59^ ^]^ The leaves have micro/nanoscale hair‐like structures along with a layer of wax crystals which significantly reduces the contact area between the water droplets and the surface of the leaves.^[^ ^60^, ^61^ ^]^ The upper epidermis of the lotus leaf provides the leaf with its superiority over other plant leaves with regard to stability, durability, and consistency as a result of its unique hierarchical structure paired with agglomerated wax tubules.^[^ ^60^, ^62^ ^]^ Water striders (*Gerris remigis*) are another example of natural water‐repellency due to hydrophobic legs as well as surface tension mechanisms, elastic momentum transfer, and wax secretion.^[^ ^63^, ^64^, ^65^, ^66^ ^]^ The WCA of the water strider's leg has been measured as >167°, and the WCA of the wax secreted by the water strider is only 105°, supporting that the surface structure must play a pivotal role in the superhydrophobicity.^[^ ^66^ ^]^ The water strider's legs consist of needle‐like hairs of hundreds of nm to 3 µm in diameter and ≈5 µm in length, protruding at an angle of 20°, and enriched with nanoscale valleys. This creates the hierarchical micro/nanosurface structure, as shown in Figure 1d, that works synergistically with the wax secretion to create superhydrophobicity.

### 1.2 Underwater Oleophobictiy in Nature

**Figure 2:**
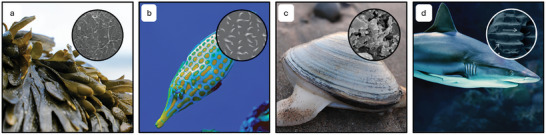
Oleophobicity in nature. a) Seaweed and an SEM image of its surface. Reproduced with permission.^[^ ^69^ ^]^ Copyright 2015, Wiley‐VCH GmbH & Co. KGaA, Weinheim. b) A Filefish and an SEM image of the hook‐like spines on its skin surface. Reproduced with permission.^[^ ^71^ ^]^ Copyright 2013, Wiley‐VCH GmbH & Co. KGaA, Weinheim. c) A Clam shell and an SEM image of the oleophobic region of its surface. Reproduced with permission.^[^ ^73^ ^]^ Copyright 2012, Wiley‐VCH GmbH & Co. KGaA, Weinheim. d) A Shark and an SEM image of its skin structure. Reproduced with permission.^[^ ^74^ ^]^ Copyright 2013, Wiley‐VCH GmbH & Co. KGaA, Weinheim.

The most abundant examples of oleophobicity in nature occur underwater, as the affinity for water facilitates the repelling of oils and low surface tension liquids. Hence, the lower part of the lotus leaf shows underwater superoleophobicity.^[^ ^67^ ^]^ The surface of seaweed displays underwater superoleophobicity that can be sustained even in high salinity and high ionic strength water environments.^[^ ^68^, ^69^ ^]^ The surface consists of pores and ridges providing a hierarchical structure (**Figure** **2**a), which works in combination with the water bonding ability of the polysaccharide surface chemistry to create superoleophobicity.^[^ ^69^, ^70^ ^]^ Fish skin and scales are often underwater superoleophobic, giving them the ability to swim through oil‐polluted waters and remain contaminant‐free. The Filefish possess oriented microscale spines resembling hooks protruding from their skin (Figure 2b) resulting in an anisotropic underwater oleophobic surface.^[^ ^71^ ^]^ The ordered structures encourage the flow of oil droplets in the head‐to‐tail direction but pin them in the tail‐to‐head direction, avoiding the advance of oil toward the head of the fish. Crucian carps have a mucus layer across a hierarchical scale structure consisting of rows of ridges (50–70 µm) across the scales decorated with small tubercles (2–3 µm) to produce underwater superoleophobicity.^[^ ^72^ ^]^ Clam shells are another example of underwater superoleophobicity, with the rough inside region using a combination of the CaCO_3_ shell composition and micro/nanohierarchical structures (Figure 2c).^[^ ^68^, ^73^ ^]^ The hydrophobic shark skin provides antifouling ability which allows the animals to swim faster as drag is reduced.^[^ ^74^ ^]^ The shark skin is seen to contain overlapping denticles with microscale riblets (Figure 2d) as well as a mucus coating.^[^ ^75^, ^76^ ^]^

### 1.3 Amphiphobicity in Nature

In air superoleophobicity and superamphiphobicity are the most demanding of the nonwetting materials. The requirements to achieve such repellency are much more strenuous and convoluted than for superhydrophobicity or underwater superoleophobicity. Accordingly, the examples in nature are much more limited. The carnivorous pitcher plant operates through a system of zones with varying wettability's: the lid, the peristome, the waxy surface of the slippery zone, and the glandular surface of the digestive zone. The lid surface has contact angles comparable to the leaves of the plant with relatively high surface free energies, with the key function of these parts being insect attachment through adhesive forces. The peristome and glandular zones are hydrophilic and oleophilic, with a very high surface energy and large polar component. The hydrophilic film causes lubricating effects, ceasing the insect adhesion. The waxy zone traps and retains the pray. The hydrophobicity of the zones has been well studied, and the hydrophilicity of the peristome and hydrophobicity of the slippery zone are understood.^[^ ^77^, ^78^, ^79^, ^80^ ^]^ However, the oleophobicity of the zones remains largely unexplored, and to the best of our knowledge is only discussed by Gorb and Gorb. They report the slippery waxy zone of the *Nepenthes alata* to have high repellency to both water and some lower surface energy liquids, namely, diiodomethane and ethylene glycol.^[^ ^81^ ^]^ In contrast to the lotus leaf effect of using microstructure cavities to block out liquids, the pitcher plant uses its microcavities to store a repellent liquid, resulting in a film over the surface and removing the air cushion, which proves ineffective against low surface tension droplets.^[^ ^82^ ^]^ When insects try to stand on this surface, the oils on their feet are repelled causing the insects to be trapped within the plant. The pitcher plants have been a fundamental influence on the design of SLIPS (slippery liquid‐infused porous surfaces).^[^ ^83^ ^]^

**Figure 3:**
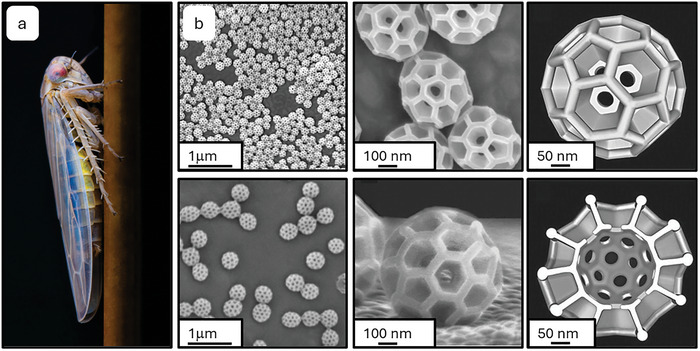
Amphiphobicity in nature. a) A Leafhopper (Athysanus argentarius). b) SEM images of agglomerated brochosomes from a Leafhopper across scales and model representations of a single brochosome and cross‐section. Reproduced with permission.^[^ ^84^ ^]^ Copyright 2012, The Royal Society of Chemistry.

Leafhoppers (Insecta, Hemiptera, Cicadellidae) have proteins arranged in the shape of hollow spheres, known as brochosomes, ≈200–700 nm in diameter creating an overall porous honeycomb‐like structure (**Figure** **3**).^[^ ^84^ ^]^ This results in repellency toward water with a W of up to 172° as well as some lower surface tension liquids including ethylene glycol and diiodomethane but not ethanol. The brochosomes are comprised of proteins and are released as colloidal suspensions which the insects spread over their skin with their legs. After the liquid has dried, the brochosomes are spread further during grooming and ultimately the insect integument becomes saturated with a thin pruinose layer of brochosomes.

**Figure 4:**
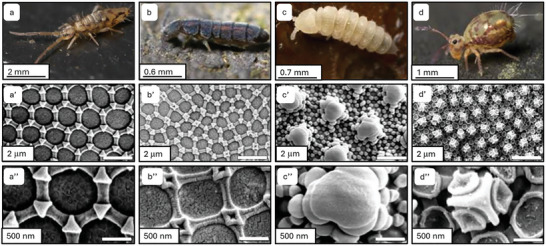
Amphiphobicity in nature. Cuticle patterns of different life forms and different patterns of Collembola. a–a″) Habitus and hexagonal structures of epedaphic Entomobryomorpha species. b–b″) Habitus and hexagonal structures of hemiedaphic Isotomidae (Entomobryomorpha). c–c″) Habitus and secondary granules with basic hexagonal structures of euedaphic Poduromorpha. d–d″) Habitus and cuticle of hemiedaphic Symphypleona. Reproduced with permission.^[^ ^88^ ^]^ Copyright 2012, Springer.

An exceptional demonstration of natural superamphiphobicity is the Springtail (Collembola) (**Figure** **4**). Springtails are skin‐breathing arthropods that live in a range of soil environments, including largely polluted areas, and the intricate skin structure and coatings provide them with antifouling ability and nonwetting characteristics facilitating their survival and ease of movement in such environments.^[^ ^85^, ^86^, ^87^ ^]^ The topography consists of an array of micro/nanoscale hexagonal or rhombic granules with overhanging re‐entrant structures.^[^ ^88^ ^]^ The structures are composed of a porous chitin base with an epicuticular layer of proteins and a homogeneous lipid layer containing groups such as steroids, esters, triglycerides, and terpenes. The cavities provide a stable Cassie–Baxter state with a high energy barrier and are nonwettable toward water, methanol, ethanol, hexadecane, and tridecane, but not dodecane or hexane.^[^ ^86^ ^]^

### 1.4 Wetting Models

Wetting models have been defined to explicitly categorize materials based on their wetting behavior. For a material to be classified as superhydrophobic/superoleophobic, it must exhibit a water/oil contact angle (W/OCA) >150°.^[^ ^89^, ^90^ ^]^ In the case of superamphiphobicity, the material must demonstrate WCAs and OCAs >150°, signifying its exceptional resistance to both polar and nonpolar substances.^[^ ^91^ ^]^ Another crucial aspect of a material's wettability is the sliding angle (SA), which describes the angle of rotation of a surface at which the liquid droplet slides off. Ideally, for superrepellent surfaces the SA is below 10°, and the lower the better. For liquid–solid systems, the solid surface energy (s) and liquid tension (γ) control the strength of interaction between the two phases, which dictates the extent of spreading and wetting of liquids over solids. The wetting behavior of materials is further classified by the wetting states: the Wenzel state and the Cassie–Baxter state.^[^ ^92^, ^93^, ^94^ ^]^ These derive from the Young's equation (Equation (1)), which describes the behavior of a droplet on a completely smooth, ideal surface.^[^ ^95^ ^]^ The equation is expressed in terms of surface tensions, where γ_sv_ is the solid–vapor surface tension, γ_sl_ is the solid–liquid surface tension, γ_lv_ is the liquid–vapor interface energy, and θ_Y_ is the Young's contact angle

(1) $$\gamma_{sv}=\gamma_{sl}+\gamma_{lv}\cos\left(\theta_{Y}\right)$$

Based on this, the theory can be developed to produce the Wenzel state.^[^ ^96^ ^]^ This describes liquid interactions with rough surfaces and can be defined by Equation (2) where θ_W_ is the Wenzel contact angle, *r* is the roughness parameter—the ratio of actual solid‐liquid contact area to the expected planar area

(2) $$\cos\left(\theta_{W}\right)=r\cos\left(\theta_{Y}\right)$$

Or, in terms of surface tensions (Equation (3)) can be used

(3) $$\cos\left(\theta_{W}\right)=\frac{r\left(\gamma_{\mathrm{sv}}-\;\gamma_{\mathrm{sl}}\right)}{\gamma_{\mathrm{lv}}}$$

The Cassie–Baxter state characterizes rough structured surfaces on which the droplets can situate upon the features, trapping air/another material pockets below, usually resulting in strong hydrophobicity due to the upward force on the droplet provided by the trapped material in the cavities. The Cassie–Baxter state can be specified by Cassie's law shown in Equation (4). Where θ_CB_ is the Cassie–Baxter contact angle and *f*_s_ is the fraction of the total surface area that is wet. In cases where there are different surface structures, each has its own fraction, *f_i_*, where the sum of *f_i_* =  1

(4) $$\cos\left(\theta_{\mathrm{CB}}\right)=rf_{s}\left(\cos\theta_{Y}\right)+f_{s}-1$$

The Cassie–Baxter state can also be portrayed with respect to the surface tension as shown in Equation (5). This equation represents systems containing air, rather than another material, between the surface and the liquid. Where γ_*i*_ indicates the respective fraction for which the corresponding energy applies

(5) $$\cos\left(\theta_{\mathrm{CB}}\right)=\sum_{i}^{n}\frac{f_{i}\left(\gamma_{i,\mathrm{sv}}\;-\;\gamma_{i,\mathrm{sl}}\right)}{\gamma_{\mathrm{lv}}}$$

### 1.5 Applications

The applications of liquid repellent materials span across a vast number of domains. Packaging is one of the largest areas for consumption of such materials. This can include packaging for food and drink, electronics, general transportation, as well as medicinal products. Also in the medical industry, surgical tools, hearing aids, and biomedical devices including catheters, stoma bags, and tubing mandate repellent properties to prevent contamination and maintain cleanliness and performance capability. Emulsifications and suspensions are also used in the medical industry as well as in cosmetics. Another application area is in electronic coatings, microfluidics, and moisture barriers to prevent short circuits. The agricultural industry relies on large scale superamphiphobic materials such as bale wrap, poly tunnels, feed bags, irrigation systems, and machinery coatings. In the case of textiles, clothing, outdoor equipment, and uniforms for hazardous environments all require superhydrophobic or superamphiphobic materials to perform their intended purpose of keeping the user dry and safe. In construction and infrastructure, bridges, building exteriors, sealants, and concretes can necessitate superamphiphobic materials to protect against weathering, water runoff, and pollutants. Smartphones, ATMs, all other displays, as well as vehicle interiors, handles, and common touch points can use oleophobicity to reduce smudging and contamination. Optoelectronics are another area where smudge resistance and antifouling are of particular interest. Self‐cleaning properties are induced by superamphiphobic materials, when liquid droplets bead and roll off a surface, they can remove debris and contaminants in the process. This is paramount for solar panels, windows, vehicles, aircraft, and spacecraft, to avoid fouling which can reduce the transparency and efficiency of these products as well as providing anti‐icing properties to enhance performance in extreme conditions. Materials with an affinity toward either oil or water and repellency toward the other are used in oil spill clean ups and environmental protection through oil/water separation, as well as coatings on marine vehicles and underwater sensors to provide antifouling and reduced drag properties allowing the vessels to operate as intended. The applications for superrepellent surfaces of all variations are extensive, and so using fluorocarbons to provide the necessary properties has caused significant detriment to the environment. A shift toward sustainable materials and methods is underway, particularly for superhydrophobic and underwater oleophobic materials. Creating sustainable superoleophobic and superamphiphobic materials invokes a more complex challenge, and with few successes on the laboratory scale, producing these materials industrially for these applications requires much more research and development as will be discussed in this review.

## 2 Current and Emerging Experimental Fabrication of (Super)Hydrophobic Materials

### 2.1 (Super)Hydrophobic Materials

Thus far, there has been significant interest in the synthesis of superhydrophobic materials along with much success. However, the focus has only recently shifted toward creating sustainable superhydrophobic materials. This means that the majority of research and current methods have significant negative environmental effects. Most representative superrepellent materials currently rely on fluorocarbon functionalities, part of a class of ≈12 000 fluorinated chemicals known as poly‐ and perfluoroalkyl substances.^[^ ^97^, ^98^ ^]^ These “forever chemicals” cause severe detriment to the environment, they are extremely resistant to degradation meaning they create perpetual waste as they come to the end of their useful life.^[^ ^99^ ^]^ They also have long biological half‐lives, which allow them to travel through the food chain and bioaccumulate, meaning even humans can ingest them in high concentrations.^[^ ^100^, ^101^ ^]^ Drinking water can also be contaminated, providing another source of ingestion for humans.^[^ ^99^ ^]^ Human ingestion of fluorinated compounds can lead to a variety of problems due to the toxic nature of fluorinated compounds, including thyroid disease, liver damage, high cholesterol, and cancer.^[^ ^102^ ^]^ Similar effects can of course be observed for other life forms, effects are particularly noticeable in decreased growth and reproduction of plant/animal species.^[^ ^103^ ^]^ Fluorine pollution is found all over the world in water, soil, and air, and due to their resistance to conventional purification and treatment processes, it is an ever‐increasing problem.^[^ ^99^ ^]^ In an attempt to help combat this, there are an increasing number of regulations being put in place by governing bodies to restrict the use of such compounds, particularly polyfluoroalkyl compounds.^[^ ^104^, ^105^, ^106^ ^]^ However, the real solution lies in completely eradicating the use of fluorinated compounds, by replacing them with sustainable alternatives that can perform to the already high set standard. This is where the challenge lies, and we can look for inspiration examples from nature, which have been refined over the course of history to provide the highest functional performance using entirely natural components.

### 2.2 Sustainable (Super)Hydrophobic Material Fabrication

In this case, the challenge of creating superhydrophobic materials that are more sustainable relies on reducing the use of fluorocarbons. As discussed, fluorocarbons cause significant detriment to the environment, and so the exclusion of such compounds will substantially increase sustainability. This can be further achieved by the ongoing search on less energy extensive methods and the utilization of readily available sustainable materials resources. Products made using natural materials, if not easily recycled, will biodegrade without releasing toxic chemicals to the atmosphere, and will not involve fluorocarbons as these are not naturally occurring materials. In fact, fluorocarbon‐free superhydrophobic materials have been relatively well researched and multiple successful designs are underway. In this section, we will summarize the most recent advances in creating environmentally friendly hydrophobic surfaces and ideas incorporated from nature.

#### 2.2.1 Sustainable (Super)Hydrophobic Material Fabrication

**Figure 5:**
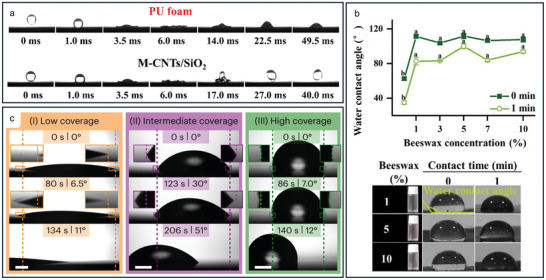
a) A water droplet being dropped onto the control PU foam surface and pinning, and onto the M‐CNTs/SiO_2_ surface and bouncing off as a result of the superhydrophobicity. Reproduced with permission.^[^ ^107^ ^]^ Copyright 2023, Elsevier Ltd. b) The WCA of the beeswax films with increasing beeswax concentration. Reproduced with permission.^[^ ^108^ ^]^ Copyright 2023, Elsevier Ltd. c) Water droplets on low, intermediate, and high coverage SAM surfaces showing time and tilt angle, with the dashed lines indicating the initial location of the droplet, displaying the wetting and sliding behavior of the materials. Reproduced with permission.^[^ ^109^ ^]^ Copyright 2023, The Author(s).

Nanotechnology was used in the form of carbon nanotubes (CNTs) to construct a sustainable superhydrophobic hierarchical CNTs/SiO_2_ and polydimethylsiloxane (PDMS) coated porous polyurethane (PU) foam.^[^ ^107^ ^]^ PDMS:n‐hexane (10:1) was used to induce superhydrophobicity into the CNTs/SiO_2_ coated PU foam, and dried, named M‐CNTs/SiO_2_PU. Various concentrations of CNTs were explored with respect to SiO_2_ nanoparticles, and 40 wt% with PDMS modification was found to be the most superhydrophobic with a WCA of 157° and WSA of 3.2°. The WCA of plain PU foam was 111.4°, and PU foam with the CNTs/SiO_2_ coating WCA was 0°, showing superhydrophilicity. **Figure** **5**a shows the water droplet being dropped on the M‐CNTs/SiO_2_ surface and bouncing, compared to on PU, the drop spread and then retracted before firmly fixing to the material. On CNTs/SiO_2_, the droplet slowly wetted the material and after 22.5 ms had almost completely penetrated the pores. This study shows a low intensity method to produce a hierarchical micro/nanostructured surface, however there is little control over the specific topography produced. The combination of two nanoparticles, SiO_2_ and CNTs improved the wear‐resistance as the two intertwined with each other, protecting the hierarchical structure when exposed to wear.

A pH indicator film was designed using a layer of sodium alginate/black soybean anthocyanin/cellulose nanocrystals with an external layer of hydrophobic beeswax.^[^ ^108^ ^]^ Without the additional beeswax layer, the film had a WCA of 77.4°, whereas with a 1% beeswax concentration, the WCA increased to 110.8° as is shown in Figure 5b. Further increase in the concentration had no tangible effect on the initial WCA, however the 5% concentration maintained the highest hydrophobicity with increased contact time, and formed a rougher heterogenous layer. Therefore, 5% was decided upon as the optimal concentration. This is a simple dispersion and casting method to develop hydrophobic films using nontoxic materials.

The molecular scale heterogeneity of a surface has been identified as an integral factor in the slipperiness of a material by determining the contact line friction (CLF).^[^ ^109^ ^]^ It was found that a low heterogeneous surface, even if hydrophilic, can exhibit low CLF due to an abundance of unconfined dynamic interfacial water molecules that facilitate the proliferation of other water molecules along the surface by providing lubricating properties. As the heterogeneity begins to increase, the molecules become confined within the hydrophilic areas, causing the previously described system to become redundant and increase the CLF. Based on this, a black silicon surface coated with Al_2_O_3_ and a SAM of octyltrichlorosilane (OTS) was designed and obtained a CLF of 0.024 µN mm^−1^. In this way, they achieved WCAs up to 109° and WSAs as low as 6°, displaying the influence of the CLF, which could be an important parameter to consider when designing superrepellent materials. Water droplets of 10 µL on low, intermediate, and high coverage SAM surfaces are shown in Figure 5c, the intermediate surface had the highest WSA and contact angle hysteresis (CAH), the low coverage surface had low CAH and the smallest WSA of 6°.

#### 2.2.2 Sustainable Biomimetic (Super)Hydrophobic Material Fabrication

The proficiency of synthetic superhydrophobic surfaces can be enhanced by integrating concepts from nature. There are many examples of durable and effective extreme superhydrophobic surfaces in nature, which have been optimized over the course of evolution. Nature has developed completely sustainable ways to produce durable superhydrophobic materials and can therefore be an important influence for designing green superhydrophobic materials. By studying these materials, the properties can be implemented to refine manufactured sustainable superhydrophobic surfaces.

**Figure 6:**
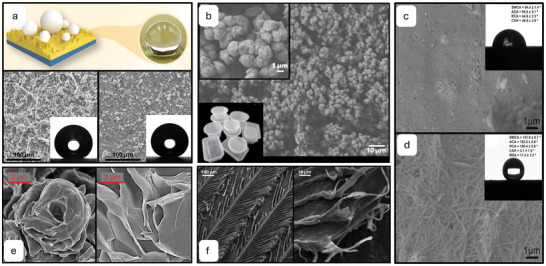
a) Illustration of the roughness causing superhydrophobicity, SEM image of the OTMS8‐CuS/CNF3 surface (left) and OTMS8‐CuS surface (right) with an inset image of a water droplet beading on the surfaces. Reproduced with permission.^[^ ^110^ ^]^ Copyright 2023, Elsevier B.V. b) SEM image of a superhydrophobic material created from waste plastic, shown in the inset. Reproduced with permission.^[^ ^111^ ^]^ Copyright 2023, Elsevier B.V. c) SEM image of unmodified PVC with an inset showing the wetting behavior. d) SEM image of superhydrophobic PVC covered with alkyl ligands and an inset showing the wetting behavior. c,d) Reproduced with permission.^[^ ^116^ ^]^ Copyright 2024, American Chemical Society. e) SEM image of the topography obtained through rice bran wax crystallization via evaporation of toluene (left) and acetone (right). Reproduced with permission.^[^ ^117^ ^]^ Copyright 2023, Wiley‐VCH GmbH. f) SEM images of the Bio‐PANI goose feather inspired material at increasing magnifications. Reproduced with permission.^[^ ^119^ ^]^ Copyright 2024, Elsevier B.V.

Inspiration was taken from rove beetles and water striders to design a superhydrophobic light‐driven actuator, employing in situ growth of copper sulfide onto a cellulose nanofiber (CNF) base followed by octadecyltrimethoxysilane (OTMS) functionalization.^[^ ^110^ ^]^ This approach combines both the Marangoni effect and vapor jet flow. OTMS induces superhydrophobicity by introducing porous, air‐trapping, micro/nanostructures, and in general, the higher % OTMS results in stronger hydrophobicity. To enhance roughness, ethanolic suspensions of various composites were spray coated onto glass, wood, and paper and dried to produce superhydrophobic structures 32 µm thick with a content of 0.3 mg cm^−2^. The surface with the highest hydrophobicity was OTMS8‐CuS/CNF3 (**Figure** **6**a) with a WCA of ≈160° and WSA of 9.80°. This means the green superhydrophobic vessel could combine Marangoni effect and vapor jet flow to allow for fully controllable motion on water, which could hold particular significance for robotic underwater sensors and surveyors.

Waste polypropylene (PP) was utilized to create superhydrophobic microspheres with hierarchical nanostructures, reminiscent of the papillae structure of the lotus leaf.^[^ ^111^ ^]^ The smooth PP surface had a WCA of 100°, crude smooth partially oxidized PP (OPP) had a WCA of 121°, and the OPP microsphere coatings had a WCA of 164° and WSA of 2.6°. The method use of using waste plastic to form hierarchical lotus leaf‐inspired surfaces was developed to work in real‐life scenarios and was tested on a wide range of sources of PP containing different additives and constituents to show the compatibility with recycled PP. Uniform microspheres with nanostructures were consistently produced, an example is shown in Figure 6b. The OPP coating was found to be superoleophilic, separating both hexane and chloroform from water, showing its potential for oil/water separation with both polar and nonpolar organic solvents. This shows an innovative use for waste PP, providing real‐world applications and solutions.

A durable superhydrophobic biomimetic microstructure coating with a porous design to reduce erosion and corrosion in gas pipelines was created.^[^ ^112^ ^]^ The design was inspired by the cylindrical microstructures of conch shells, and consists of a 316L steel substrate, followed by a PDMS layer, made porous using citric acid monohydrate, then a 3D printed biomimetic layer of metallic cylinders (≈300 µm tall, ≈300 µm spacing). This layer was then coated with epoxy resin (EP) to exuberate the attachment of the following layer, a solution of lauric acid (LA)‐modified TiO_2_ nanoparticles and CNTs dispersed in water. The CNTs discourage the aggregation of nanoparticles and reinforce bond strength between EP and nanoparticles, and the LA‐modified TiO_2_ nanoparticles give texture control and lower surface energy resulting in a WCA of 154.4° and WSA of 3.8°. The PDMS sponge layer maximizes mechanical resistance by absorbing impact and acting as a spring. A combination of experimental and computational studies allowed for the design measurements to be decided as optimal compared to designs inspired by pangolin skin (triangular prism), crocodile skin (cuboids), or python skin (quadrangular prism), for the lowest stress whilst maintaining superhydrophobicity and be carried forward into the further studies.

3D printing was also used to create a floating origami zigzag structure inspired by the superhydrophobicity of water striders and the air‐trapping capability of diving bell spiders based on a zigzag structure.^[^ ^113^ ^]^ The apex angle of the zigzag structure was varied through 30°–150°. The 3D printed polylactic acid (PLA) surface was then briefly immersed in a CH_2_Cl_2_ and silica dimethyl silylate (R972) containing solution. The CH_2_Cl_2_ corroded the substrate creating microholes, which were then occupied by the superhydrophobic R972 particles. The trapped air drastically promotes the floating ability of the structure compared to the flat sheets that depend entirely on the superhydrophobic coating for buoyancy. The best‐performing structure was the 30° apex angle structure, and exhibiting a WCA of 146°. As the apex angle increases, the increase in buoyancy decreases as the sealed air decreases. To further improve the air trapping capability, horizontal cross walls were added to the structure. This new perpendicular division splits the trapped air segments into smaller areas the cross walls can counterbalance the longer air sealing distance at higher angles and improve the floating ability as well as the reliability of the structures. This green design shows potential in applications such as cable management that require a durable buoyant structure. It is evident that the smaller apex angles perform much better, and cross walls significantly enhance the materials.

3D printing is a remarkably tuneable technique, which allows precise and convoluted structures to be accurately produced. This is ideal for systematically identifying trends and structural effects on properties. However, there are limitations; the most common materials for 3D printing are plastics or resins, which come with environmental issues, although it is also commonly used with PLA, which is made from renewable sources such as corn starch and therefore is biodegradable. As well as this, 3D printing is being increasingly implemented with more biobased materials.^[^ ^114^ ^]^ The dimension limitations are another drawback of 3D printing as a technique for bioinspired liquid repellency. Different feature sizes require different machines or nozzles, meaning in order to proportionately mimic the hierarchical structures found in nature a combination of printing technology would be required to incorporate macro‐, micro‐, and nanoscale structures. This complexity increases the cost and time requirements of the process.

A biodegradable, biobased, superhydrophobic, corrosion resistant, graphene‐doped polylactic acid (G‐PLA) aerogel with a honeycomb resemblant pore structure and vertical channels using a directional freezing technique was designed.^[^ ^115^ ^]^ Graphene donates its incredible photothermal ability to the aerogel and increases the diameter of the channels as well as the total volume and porosity. The pore area increase is favorable for the absorption of oil. As well as this, the G‐PLA aerogel was extremely ductile, showing no cracks or deterioration after bending, stretching, and twisting tests. It also possessed good thermal stability, under TGA and displayed similar combustion properties to the melamine sponge deeming it sufficiently stable for marine oil spill clean‐up. Both PLA and G‐PLA present WCAs over 150°, and G‐PLA retains superhydrophobicity after immersion in pure water, seawater, alkaline, and acidic solutions for 7 days, as well as across a range of pH's whilst being heated from 20 to 80 °C. The superhydrophobic G‐PLA aerogel shows good stability and exciting solar‐enhanced oil–water separation ability, whilst being fully biobased and easily synthesized. The directional freezing technique produces an ideal channeled structure for oil/water separation.

A superhydrophobic coating for polyvinyl chloride (PVC) food packaging utilizing a polydopamine (PDA) SAM was designed, inspired by the underwater adhesion of marine mussels as well as nanodiamonds, and alkyl silanes.^[^ ^116^ ^]^ The initial PVC had a WCA of ≈84.4° and the final product exhibited a WCA of ≈152°, WSA of ≈11.3°, and CAH ≈3.1° as shown in Figure 6c,d. Octadecyltrichlorosilane (ODTS) introduced a network of 10–100 nm diameter, micrometer length fibers to the surface physically adsorbed to the PDA. The roughness ratio was increased from 1.04 for PVC to 1.36 for the final superhydrophobic surface. The antibacterial properties were investigated, and the sample showed a 99.2% decrease in the density of fixed *Escherichia coli* and a 99.3% reduction in the density of fixed *Salmonella* *enterica* cells. The substrate was saturated with food grade surfactant and did become wetted. However, after rinsing with water the superhydrophobicity was regained, showing the product's ability to be washed in applications. Overall, a green coating was synthesized, providing superhydrophobicity as well as antibacterial properties, ideal for applications within the food industry. SAMs provide many benefits as a synthesis method. The thickness of the layer can be specifically controlled, the process is inexpensive, energy efficient, usually uses readily available materials, with minimal waste, which can be functionalized to meet requirements, and are often durable and stable due to the strong bonding. As a result, it is a promising technique for industrial scale production of sustainable superhydrophobic materials.

Simple approaches to creating superhydrophobic coatings from natural waxes by solvent evaporation or straightforward drop coating of molten waxes, inspired by the lotus leaf, were systematically investigated.^[^ ^117^ ^]^ The majority of the study focused on using toluene and acetone as solvents with rice bran wax (RBW), which had a benchmark smooth surface WCA of ≈109°. Solvent evaporation was seen to produce much more varied topography (Figure 6e), with toluene evaporated RBW creating uniform microroses of ≈5 µm diameter with petals of ≈100 nm thickness. The induced roughness amplified the contact angle to 147°–150° however the droplets were pinned to the surface and unable to roll off. Acetone evaporates much quicker than toluene resulting in high supersaturation, and so elicits a nonuniform surface of deformed petals of ≈100 nm thickness and ≈1 µm maximum peak height configured in random orientations and an overall irregular surface. Despite the lack of homogeneity, the smaller size of the features further increased the WCA to ≈160° with WSAs of less than 5°, exhibiting a self‐cleaning effect akin to the lotus leaf. Binary mixtures of toluene and acetone were also used, as the concentration of toluene increases so does the solubility and the solvent vapor pressure and crystallization rate decrease. The weight % of acetone in the solution was varied, and below 25% the droplets were pinned to the surface. At 50%, the surfaces were similar to those formed from simple cooling of molten waxes. The highest WCA and lowest WSA were still that of the 100% acetone solvent evaporation. Acetone‐evaporation produced the highest WCAs and lowest WSAs for all waxes tested in the study, with WCAs over 150° and WSAs ≈3°, apart from soy wax. As well as this, all waxes formed sticky surfaces from toluene‐evaporation. From this study, we can assume that solvent evaporation will consistently increase microroughness, and solvent/temperature can be varied depending on the desired outcomes. This method is extremely straightforward and energy and material efficient. Depending on the durability and stability of the materials, which was not studied in depth, this simple solvent evaporation method could hold significant purpose in industrial sustainable superhydrophobic material production.

A simple spray method micro–nanostructured, superhydrophobic coating inspired by the lotus leaf was created.^[^ ^118^ ^]^ The coating consisted of bisphenol‐A EP, epoxy‐modified silicon resin (SR), and sometimes expandable graphite (EG) and/or a cyclodextrin‐based flame retardant “MCDPM”. When EG and MCDPM occur in the same coating (EP/SR‐3), they work synergistically to introduce micro/nanoscale roughness and lower the surface energy to produce superhydrophobicity exhibiting a WCA of 153.9° and an WSA of 8° with a surface roughness (Ra) value of 147.2 µm. This coating was also repellent toward cola, tea, milk, and coffee. The simply fabricated, scalable, bioinspired, sustainable coating induces steel with superhydrophobicity as well as enhanced properties such as thermal insulation with various potential applications in building, equipment, or vehicle industries.

Biomass carbon (BMC) was incorporated into a polyaniline (PANI) coating, replicating the structure of goose feathers through nanocasting to produce a superhydrophobic, anticorrosion, antibiofilm, biomimetic surface as shown in Figure 6f.^[^ ^119^ ^]^ BMC was formed through calcination of agricultural coconut waste, providing a valuable use of a waste product, in a simple, effective, and industrially scalable nanocasting method. In order to recreate the surface structure of natural goose feathers, which comprises micrometer rachis adorned with nanorange barbules, PDMS molds were used to create negative impressions of the goose feathers. Then PANI/PANI‐1 solutions were cast into the molds to create Bio‐PANI and Bio‐PANI‐1, respectively. The BMC had a surface area of 3.071 m g^−1^ and pore size of 43.52 nm, classifying it as a mesoporous material likely to possess gas barrier properties and moisture resistance. The WCA of BMC alone was found to be ≈133°, PANI was ≈93°, PANI‐1 was ≈98°, Bio‐PANI was ≈145°, and Bio‐PANI‐1 was ≈153°. The contact angles show that superhydrophobicity was induced as a result of a synergistic effect of the biomimetic goose feather structure alongside the structure and composition of BMC and hydrophobic, nonpolar nature of the phenolic rings comprising PANI. Bio‐PANI‐1 provides a useful application for agricultural waste into a facile green process to synthesize anticorrosion, antibiofilm, superhydrophobic materials with potential applications in a range of areas such as in marine vessels and the food industry.

## 3 Current and Emerging Experimental Fabrication of (Super)Oleophobic Materials

### 3.1 (Super)Oleophobic Material Fabrication

As mentioned above, the most established route to creating superoleophobic surfaces involves the use of low‐surface energy fluorinated compounds in combination with hierarchical micro/nanostructures. Rough surfaces usually still require a fluorinated layer in order to exhibit superoleophobicity, whereas hierarchical double re‐entrant structures may not.^[^ ^120^ ^]^ This is demonstrated clearly by using electrospinning to synthesize various polyvinylidene fluoride (PVDF) nanofibrous membranes (NFMs).^[^ ^121^ ^]^ The surface roughness of the membranes was varied to produce a relatively smooth surface, a microstructured surface, and a nanostructured surface. In certain cases, fluorinated polyhedral oligomeric silsequioxane was added to the initial PVDF solution, inducing superhydrophobicity in the resultant membrane. The membranes were tested for several properties relating to their potential to reduce mineral scale accumulation on reverse osmosis seawater membranes and elevate desalination, summarized in **Table** **1**. Electrospinning can be used on a variety of polymers and materials, with fine control over the fiber dimensions and customizability over any functional groups. Electrospinning is already used on an industrial scale and can be a simple way to produce nanofibers but naturally requires high voltage, potentially toxic solvents. The produced materials may be nonuniform or fragile, requiring additional cross‐linking steps to improve durability and potentially addition of adhesives or other additives to produce a usable film or coating.

**Table 1:** A summary of the key properties and findings of smooth, microstructured, and nanostructured PVDF NFMs.

	Smooth NFM	Microstructured NFM	Nanostructured NFM
Water contact angle [°]	128	153	163
Oil contact angle [°]	–	128	134
Liquid entry pressure [bar]	1.17	1.39	1.51
Air permeability	Superior	Diminished	Diminished
Water flux at 60 °C (L m^−2^ h^−1^)	56.59	52.36	48.05
Desalination water recovery at 3.5 wt% NaCl [%]	60.15	s	90
Signs of NaCl after usage	Penetration in cross‐section Crystals on the surface	Penetration in cross‐section Flake crystals on the surface	No traces in cross‐section Minimal crystals on the surface
Hydrophobic recovery after use (water contact angles, °)	112	134	152

An oleophobic surface was developed by heterogeneously bonding PDMS with micropillars and photoresist SU‐8 caps creating doubly re‐entrant structures, with the ability to restore their shape after extreme mechanical deformation and to repel a variety of liquids.^[^ ^122^ ^]^ The fabrication technique used soft photolithography in combination with curing PDMS on silicon or SU‐8 molds, which were coated with a hydrophobic coating of trichloro(1H,1H,2H,2H‐perfluoroctyl)silane. The resultant structure consisted of PDMS pillars of 20–30 µm in height and 30 µm in diameter, topped with SU‐8 caps of 7 µm height and 50 µm diameter, with a 2 µm thick overhang with a height of 5 µm. The WCA, advancing, and receding angles were measured to be ≈156°, ≈167°, and ≈118°, respectively. The isopropanol contact angle, advancing, and receding angles were 130°, ≈159°, and ≈109°, respectively. The structures proved to be reasonably robust, maintaining a high WCA even after repeated deformation, indicating a scalable fabrication route for resilient, and repellent surfaces.

**Figure 7:**
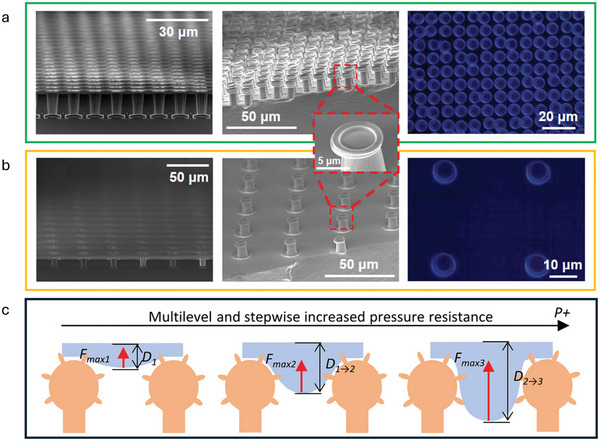
a) SR1 silicon master, SEM, and optical microscope images of the fabricated SR1 structures (left to right). b) SR6 silicon master, SEM, and optical microscope images of the fabricated SR6 structures (left to right). Reproduced with permission.^[^ ^123^ ^]^ Copyright 2020, American Chemical Society. c) Schematics of the multilevel resistance to liquid intrusion by the armored re‐entrant structures. Reproduced with permission.^[^ ^125^ ^]^ Copyright 2023, Wiley‐VCH GmbH.

Another hierarchical superamphiphobic PDMS surface was developed through photolithography, inspired by the square, concave top, pillar array displayed by the Springtail, *Tetrodonto phorabielanensis*.^[^ ^123^ ^]^ Once again, PDMS was cured into a negative C_4_F_8_‐coated silicon mold with double mask align methods. Polystop etching and concentric etching proved vital in eliminating any defects in the structure of the silicon master. Two different microstructures arrays were created, with variation in the pillar spacing, for the surface named SPM1, the interpillar spacing was 5 µm, created using silicone master SR1 (**Figure** **7**a) and for the surface named SPM2, the interpillar spacing was 30 µm, created using silicone master SR2 (Figure 7b). The WCA for the SR1 surface was over 150°, however mineral oil on the surface existed in the Wenzel wetting state. The SR6 surface also displayed a WCA of over 150° as well as a mineral OCA of over 150°. Furthermore, theoretical calculations were performed, and these results along with experimental results, showed that the WCAs and OCAs are seen to increase with the SR values of pillar spacing. The advantage of these methods is the ability to reuse the negative silicon master to produce durable and resilient materials, however the use of fluorocarbon coatings and certain photolithography processes come with negative environmental impacts.

Similarly, a superamphiphobic flexible material was designed through a single‐exposure UV proximity soft lithography method resulting in re‐entrant topography inspired by the Springtail.^[^ ^124^ ^]^ During the lithography, the photomask had a coaxial circular aperture and ring, when exposed to UV this created a positive mold of capped‐pillar re‐entrant structures. This then underwent fluorosilanation and elastomeric polymer mold curing to create a soft negative replication. The fluorination step was performed again which not only enhanced the repellency but also assisted in the demolding by reducing the risk of fractures or deformations of the final material. One of the inhibiting factors of using molds to create re‐entrant structures is the difficulty of the demolding step. In this case, this could only be overcome with the use of fluorocarbons, which negates the advantages of the re‐entrant structure providing repellency without the need of chemical modification. The focused‐on surface had features ≈60 µm in height, with a pillar thickness of ≈70 µm, cap diameter of ≈34 µm, and spacing of 364 µm. This material exhibited a WCA of 154.7° and OCAs over 150° for ethylene glycol, olive oil, 50 wt% ethyl alcohol, and hexadecane. To reach smaller size features, a thermoresponsive hydrogel, poly(*N*‐isopropylacrylamide) (PNIPAM), was used to create a shrunken surface. The obtained shrunken surface introduced microscale wrinkles to the surface, causing a three‐layer hierarchy with the fabricated structures and fluorosilane nanoparticles. The capped pillars now had a height of ≈24 µm, pillar diameter of ≈15 µm, cap diameter of ≈25 µm, and spacing of ≈131 µm. The shrunken surface had a higher pressure resistance and improved robustness. PNIPAM or other thermoshrinking hydrogels show potential for fabrication of sub‐micrometer scale structures through soft lithography and could provide an approach toward smaller scale topographies, closer to those observed in nature.

An amphiphobic surface was designed, composed of a micrometric re‐entrant structure as well as a nanometric re‐entrant structure, named armored re‐entrants (AR), enabling both minimal solid–liquid contact and liquid pressure resistance, mimetic of the hierarchical structure of the Springtail (Figure 7c).^[^ ^125^ ^]^ The surface's superrepellent properties were able to withstand knife scratching, ultrasonic cleaning, pH changes, and temperatures up to 300 °C, and last for over 120 days. The AR surface also possesses biological and medical application potential as it has proved to effectively prevent the colonization of bacterium. Even though the surface was bioinspired, there was still a fluorination step using perfluorodecyltrichlorosilane to decrease the surface energy and create a WCA of >150° and OCA >140°, meaning there are still implicit detrimental environmental effects. The structures were produced using nanosecond lasering, which is an extremely scalable approach applicable to a wide range of substrate materials, creating re‐entrant structures without the need of chemical use for etching or additives for creating a film or coating.

A PU synthetic leather surface was dip coated with fluorine‐functionalized nanosilica particles (FNPs) to induce superamphiphobicity.^[^ ^126^ ^]^ The resultant material showed self‐cleaning properties with reasonable thermal and mechanical stability. Bacterial adhesion was reduced by over 98% for *E. coli* and *Staphylococcus epidermidis*, compared to the control surface of unmodified PU. On the control synthetic leather surface, the WCA was ≈79.3° and OCA was less than 10°. After the FNPs coating procedure, these angles had increased to ≈161.5° and ≈152.2°, respectively. The material also showed effective self‐cleaning properties using both water and oil droplets. The tape peel test was performed, after which the material maintained its self‐cleaning ability, suggesting mechanical stability. The superamphiphobicity was also retained after being exposed to UV treatment, indicating photostability.

### 3.2 Sustainable Biomimetic (Super)Oleophobic Material Fabrication

As discussed, the most prominent cause of unsustainability within superoleophobic surfaces is the rampant use of fluorocarbons. Here we explore recent advancements without the use of fluorocarbons to address the climate issues. Biomimicry has also been invaluable in designing sustainable underwater superoleophobic surfaces. With plentiful examples in nature, many ideas and concepts are provided and can be exploited for constructing green underwater superoleophobic surfaces. Underwater oleophobicity is more simplistic than in air oleophobicity as it can use hydrophilicity to reduce the affinity toward oil and so such materials are relatively straightforward to design and manufacture. Biomimicry has inevitably been used to create green underwater superoleophobic surfaces. For example, Oh et al. developed an eco‐friendly fish‐skin biomimetic membrane for oil–water separation, with a WCA of <5°, allowing for permeability, and an underwater OCA of 160°–170° with the use of the bead‐on‐string layer of sub‐micrometer particle and nanofibrils.^[^ ^127^ ^]^ The membrane was able to separate floating oil with an efficiency >99%, as well as separating dispersed and emulsified oils. The membrane was composed of a fish gelatin nanofiber lower layer cross‐linked with a bead‐on‐string upper layer, deposited by electrospraying. The entire process from manufacture to end of life is environmentally friendly and nontoxic, providing a promising approach for oil–water separation.

Another underwater superoleophobic material, showing robustness and transparency, was produced by spreading a biomimetic mineralized chitosan solution presenting hierarchical nanostructures alongside a high‐energy inorganic CaCO_3_ aragonite, inspired by columnar nacre.^[^ ^128^ ^]^ Amorphous precursor calcium and carbonate nanoparticles were used rather than ions, replicating the natural biomineralization procedures found in nacre. The coatings were used to create meshes, which consistently showed efficiencies of over 99% for oil/water separation with a variety of oils. Again, this has potential applications in microfluidic devices as well as antifouling, underwater optics, oil–water separation, etc. The film must be homogenous to achieve transparency and extreme pH's will degrade the mineral film, however this can be restored via repeating the mineralization step.

Taking inspiration from nature is even more important regarding in air superoleophobic material design. As superoleophobicity inherently gives rise to more challenges than the other materials discussed, this area is still in its infancy, and it is important to learn from organisms that have mastered this property. We have seen the well‐studied, successful examples in nature—Collembola and Leafhoppers. Research into these natural examples has begun, but natural materials often lack the durability and performance of fluorocarbons and oil‐repellent surfaces rely heavily on re‐entrant curvatures which can be demanding to create on sustainable materials and through sustainable methods. So, biomimicry is pivotal in the search for these materials. Further sustainable superoleophobic material research is needed. By learning from these species, it should be possible to provide a life‐changing, sustainable solution to the current harmful methods.

Helbig et al. investigated the skin patterns of Springtails; these arthropods have evolutionarily developed wetting resistant, antiadhesive skin.^[^ ^85^ ^]^ The patterning consists of either a hexagonal, rhombic, or hexagonal and rhombic mesh of interconnected primary granules with a negative curvature, which enforces an energy barrier upon any impeding liquid. This results in superhydrophobicity, with contact angles over 160°, as well as superoleophobicity, resisting wetting against tridecane, chloroform, and hexadecane, without the need for fluorocarbons. Even up to a pressure of 3.5 atm the skin remains superrepellent. The nanoscale patterning of the Springtail skin is much smaller than synthetic surfaces explored thus far, with the primary granules having side lengths of 200–300 nm. Additionally, gram‐positive bacteria, gram‐negative bacteria, and fungi were presented to the Springtails under standard culture conditions for four days, and there was no detectable change to the skin surface. The Springtail skin provides a riddle of instructions on how to create a green, durable, superoleophobic material and will be a vital tool as the research continues.

**Figure 8:**
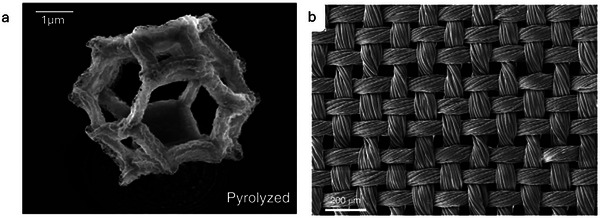
a) SEM image of the nickel nanolattice structure after pyrolysis. Reproduced with permission.^[^ ^129^ ^]^ Copyright 2018, The Author(s). b) SEM image of the hydrophobic fabric. Reproduced with permission.^[^ ^131^ ^]^ Copyright 2020, Springer Nature Limited.

A regime was developed to produce nanoarchitected metal structures, similar to the structure of the Leafhopper, through two‐photon lithography to produce computer‐designed structures followed by pyrolysis in an inert atmosphere at 1000 °C and then a reducing atmosphere at 600 °C to shrink the structures.^[^ ^129^ ^]^ In this way, beams of 300–400 nm were produced with a 20 nm mean grain size, as seen in the lattice in **Figure** **8**a. The nanolattices could withstand 6.9–18.2 MPa of compression before the first buckling occurred, and the stiffness of the nanolattices was found to be 47–174. The strengths of the lattices are found to be less susceptible to the expected decrease in strength with decrease in beam diameter, showing the influence of the Leafhopper inspired lattice structure. This is a promising technique to create nanoscale structures like those seen in nature and is a very interesting area for further research into the effect of such nanostructures on wettability, as well as their ability to be used in coatings. Another area of interest is the shrinking method, as this could be applied to other microstructures to obtain nanostructures with improved repellency properties.

Inspired by the *Manis pentadactyla*, microcomposite and micro–nanocomposite biomimetic structures on Al_2_O_3_/TiC ceramic substrates were prepared by nanosecond lasering and followed this with an ODTS surface modification.^[^ ^130^ ^]^ The research aimed to create a ceramic that could be used to obtain high transport speed and cutting performance in vegetable‐oil‐based minimum quantity lubrication interrupted coating. The oleophobic zone, known as *Z*_O_, featured square protrusions as its microstructure and showed in air oleophobicity of ≈115°.

In the textiles industry, perfluorinated systems have so far been the only way to obtain consistent nonzero contact angles with hexadecane and lower surface tension liquids, needing to overcome the limited porosity of textiles, especially for close‐packed fibers. This was studied to develop a systematic design process to create perfluorocarbon‐free oleophobic textile materials.^[^ ^131^ ^]^ It was found that adding a second layer of finer texture bypasses the limited porosity issue, which can increase the contact angle according to the Cassie‐Baxter state, as the amount of air in contact with the liquid will increase. The porosity of the textile was related to the chemistry to describe combinations required to achieve oleophobicity. It was found that PDMS would need a porosity of 4.7 ≥ 5.4. This is a precise porosity which explains why it is difficult to achieve. A nylon fabric was treated with O_2_ plasma, dip‐coated into a silanol solution and cured in an oven. It was then plasma treated again and exposed to 1,3‐dichlorotetramethyldisiloxane vapors. The resultant fabric (Figure 8b) displayed contact angles of over 90° to water, artificial sweat, castor oil, olive oil, and canola oil, but only ≈33° for hexadecane. Similarly, a low‐cost, lignin‐based coating for application to cellulose fabric, imparting superhydrophobicity, as well as increased textile favorable properties such as UV protection, softness, and crease‐recovery was devised.^[^ ^132^ ^]^ The lignin was obtained from agricultural straw waste and functionalized with 3‐glycidoxypropyltrimethoxysilane and a silicon‐based softener to finalize the hydrophobic coating. The highest WCA, 157.2°, was achieved with 2.0% w/v lignin, in comparison to the control coating which had a WCA of 54° after 12 s and 0° after 60 s. The 2.0% w/v lignin coating was also repellent toward polar solvents including ethylene glycol (CA ≈ 170°), glycerin (CA ≈ 165°), and hydrazine (CA ≈ 146°), but not toward nonpolar solvents such as toluene, DMF, n‐hexane, or n‐hexadecane.

Zhang et al. created double re‐entrant superamphiphobic flexible surfaces through a one‐step process, without the use of fluorination, inspired by the Springtail.^[^ ^133^ ^]^ The features were developed using photoresist templates, and the negative templates were fabricated using two layers of photoresist and repeated exposure before PDMS casting was performed. Various sizes of the capped pillar structures were investigated, and in order for successful demolding the chosen size was a cap thickness of 2 µm and a pillar diameter of 10 µm. The Cassie‐Baxter equation was used to optimize the feature spacing based on the solid fraction area being less than 0.067. This meant an overhang height of 2 µm, cap diameter of 21 µm and an interpillar spacing of over 89 µm. The material had contact angles over 150° for water, glycerol, ethylene glycol, vegetable oil, ethanol, and 3M Fluorinert FC‐70, which has a surface tension of just 18 mN m^−1^. On a flat PDMS surface, this liquid had a contact angle of <10° and on a single re‐entrant version could also not prevent wetting, showing the incredible impact of the double re‐entrant features with optimized dimensions. Droplets of ethanol were released onto the surface and bounced completely back off, expressing the nonpinning repellency of the material. Generally, oxygen plasma treatment renders a material hydrophilic, however, after a few hours in ambient conditions, the material was able to recover its superhydrophobicity. The material could maintain its properties for ≈2000 stretching cycles and ≈10 000 bending cycles, displaying its mechanical strength for use in industrial applications such as antiwetting adhesives, self‐cleaning surfaces, or drag reduction.

A Fresnel aperture diffraction modulation approach was used by the same research group in microscale lithography with a molding process to produce a sustainable flexible double re‐entrant superamphiphobic material, inspired by the Springtail.^[^ ^134^ ^]^ The features possess nanoscale overhangs, the negative mold of which was created in a single‐layer photoresist, which is the first time this has been reported. Based on calculations and production of the template, development time, UV dose, and aperture distance and diameter controlled the resultant topography. After various calculations based on the Cassie‐Baxter equation and breakthrough pressure, the optimized dimensions were chosen as a pillar height of 30 µm, cap diameter of 21 µm, cap thickness of ≈3 µm, overhang height and width of ≈500 nm, angle between overhang and horizontal plane of 72°, and a pillar spacing of 80 µm. No further treatment was applied to the PDMS material, and it showed contact angles over 150° for water, glycerol, ethylene glycol, vegetable oil, 50 wt% ethanol solution, and ethanol. The contact angle for ethanol was ≈153°, compared to 22.5° on the flat PDMS control surface. Ethanol was seen to bounce on the double re‐entrant material when dropped from a height, showing the pressure resistance of the material. Even after oxygen plasma treatment, the structured surface remained superhydrophobic. The material also remained superamphiphobic after 2000 stretching cycles and 10 000 bending cycles, showing promise for applications in self‐cleaning, anti‐biofouling, or drag reduction industries.

The use of photolithography is the most successful method to produce superoleophobic materials without fluorocarbons. However, it may not be considered sustainable due to the high energy requirements and specific substrate materials as well as the use of various hazardous gases. It is also not feasible, efficient, or cost effective for industrial production of these materials for their commercial applications. Despite these limitations, the technique provides an avenue to tangibly identify the benchmarks and parameters required for superoleophobicity, which can then be recognized in the search for industrially scalable, energy efficient, low resource intensive, and fluorocarbon‐free production methods. It allows for precise control over the dimensions of the structures, as well as accurate designs of patterns that is unachievable with other methods. This means variables such as the CLF and feature dimensions can be systematically explored. Furthermore, the limitations of photolithography can be minimized when using the technique to produce a stencil and then performing casting with a material such as PDMS as seen in previous examples. In this way, providing demolding is achievable, the materials can be continuously produced from only one photolithography process, reducing the impact of the intensity of photolithography per unit fabrication and increasing the scalability of the method.

## 4 Computational Modeling Methods in Surface Design

Advances in supercomputing, cloud computing, and graphics processing units (GPUs) alongside atomistic modeling, high‐throughput computing, and artificial intelligence are revolutionizing the way we design and develop materials and even the way we understand the world. Computational and digital tools have entered almost all scientific disciplines, as well as our lives. Researchers are taking advantage of these computational tools to accelerate molecular and materials discovery and to understand fundamental mechanisms of matter at time and length scales beyond experimental techniques capabilities, e.g., atomistic level interactions and multivariable optimization processes.

**Figure 9:**
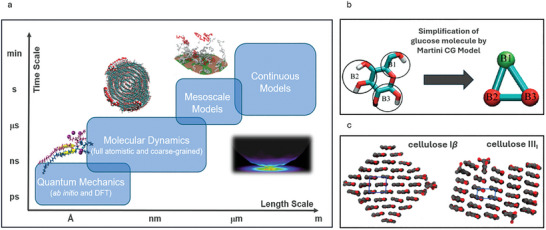
a) Classification of computational modeling approaches based on time and length scale. Reproduced with permission.^[^ ^135^ ^]^ Copyright 2019, American Chemical Society. Reproduced with permission.^[^ ^136^ ^]^ Copyright 2011, American Chemical Society. Reproduced with permission.^[^ ^137^ ^]^ Copyright 2021, American Chemical Society. Reproduced with permission.^[^ ^138^ ^]^ Copyright 2018, American Chemical Society. b) Representation of simplified glucose molecule by Martini CG Model. Reproduced with permission.^[^ ^142^ ^]^ Copyright 2009, American Chemical Society. c) Representation of cellulose I_β_ and cellulose III structures by Martini CG Model. Reproduced with permission.^[^ ^143^ ^]^ Copyright 2015, American Chemical Society.

In computational multiscale modeling and simulations, methods include ab initio methods, density functional theory (DFT), fully atomistic molecular dynamics (FA‐MD), coarse‐grained molecular dynamics (CG‐MD), and finite element methods (FEMs). **Figure** **9**a summarizes the traditional view of multiscale modeling and simulations applied across length and time scales.^[^ ^135^, ^136^, ^137^, ^138^ ^]^

Quantum mechanical methods like DFT provide information regarding electron‐related properties and processes within molecules and materials, e.g., total energy, band structure, and magnetic properties of the material. Its computational demands, however, limit the use of DFT methods to systems with a few hundred of atoms. MD tries to address such limitations, reaching systems with few nanometers in length and nanoseconds in time scale.^[^ ^139^ ^]^ In FA‐MD simulations, the behavior of electrons is neglected, and the quality of results relies on interatomic potential functions and parameters, i.e., force fields.^[^ ^140^ ^]^ The validation of the force field can be performed by quantum mechanical calculations or based on experimental data.^[^ ^139^, ^141^ ^]^ FA‐MD gives information about the relationship between the structure and its physical characteristics, such as the radius of gyration and gel transition temperature. CG‐MD simulations are used to study molecular systems for longer time scales at a more acceptable computational cost. One of the most common CG models is the Martini model. A representation of glucose with the Martini model as beads is shown in Figure 9b.^[^ ^142^ ^]^ Representations of cellulose in the Martini model as cellulose I_β_ and cellulose III structures are shown in Figure 9c.^[^ ^143^ ^]^

To achieve larger systems on the macroscopic scale, FEM is applied to problems such as structural mechanics, heat transfer, fluid flow, or electromagnetic fields as long as the problem is stated by proper differential equations and boundary conditions. The FEM method is used in computer‐aided engineering, such as automotive applications, manufacturing process simulations, electrical and electronics engineering applications, and aerospace engineering.^[^ ^144^ ^]^

**Figure 10:**
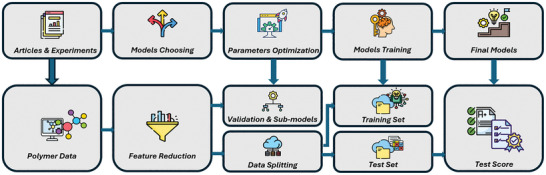
Schematic representation of ML modeling. Reproduced with permission.^[^ ^147^ ^]^ Copyright 2024, American Chemical Society.

Besides these computational modeling approaches, artificial intelligence (AI) and especially machine learning (ML) are revolutionizing our ways of designing, modeling, and simulating materials and molecules. As large quantities of data are acquired in computational processes, it makes the analysis, interpretation and prediction of data extremely complex. ML allows researchers to design new materials and to predict properties beyond capacity.^[^ ^145^, ^146^ ^]^ An example of an ML‐driven materials modeling scheme is shown in **Figure** **10**.^[^ ^147^ ^]^

However, even with the use of ML, the challenge is at the mesoscale; atomistic models are too computationally demanding to simulate systems that large, while FEM lacks the level of detail required to capture mesoscale material features and processes. This is a persistent challenge in multiscale modeling, which also affects the application of these methods to bioinspired materials designs with tailored surface properties. Nevertheless, with their limitations, these methods have proved helpful as described below.

Computational tools, including DFT, MD, FEM, and new ML‐based methods, have already proven valuable in material development in areas such as metal‐organic frameworks, semiconductors, catalysis, and the design of materials with specific properties such as superhydrophobicity for underwater applications or biosensing.^[^ ^148^, ^149^, ^150^, ^151^, ^152^, ^153^ ^]^ FEM and numerical methods are mostly used in engineering applications, where the systems under study are usually too large for atomistic simulations. These include fluid dynamics and heat transfer, although some work in energy or agriculture could also include DFT, MD, and ML.^[^ ^154^, ^155^, ^156^, ^157^, ^158^, ^159^, ^160^ ^]^ DFT and MD are traditionally more suitable to understand protein dynamics and their interactions,^[^ ^161^, ^162^ ^]^ as well as drug design, although they are now being combined with ML for effective disease diagnosis, or tissue engineering.^[^ ^162^, ^163^, ^164^, ^165^, ^166^, ^167^, ^168^, ^169^, ^170^, ^171^, ^172^, ^173^ ^]^ Moreover, all these computational tools provide information regarding natural hierarchical designs, like the self‐assembly behavior of fatty acids for the formation of protective cutin biopolyester layer in plants, the fracture mechanism of chicken eggshells showing resistance from outside but breaking easily from inside or the wettability of hair surfaces with different damage ratios that inspired the development of hydrophobic packaging materials, sports safety equipment, and new cosmetic formulations.^[^ ^174^, ^175^, ^176^ ^]^ Another example is the study of the hardness to stiffness adaptability of the jaw of the marine worm, i.e., Nereis Virens.^[^ ^177^ ^]^ Similar tools have been used to develop water harvesting materials inspired by the hydrophilic structure of desert beetles for water drop nucleation and the hydrophobic surface of lotus leaves for water removal and transport.^[^ ^178^ ^]^ Using gecko‐inspired topology on stainless steel, adapting the roughness property of coral‐reef structure to silica coating varnished surfaces, bionic flexible pressure sensor inspired by a curved Mantis leg, or NO_2_ sensors inspired by a dog's nose are additional examples of bioinspired materials developed with the assistance of MD or FEM.^[^ ^179^, ^180^ ^]^ It is also possible to use similar MD and FEM computational methods for the development of bioinspired materials showing improved mechanical properties.^[^ ^181^ ^]^

In superhydrophobic and superoleophobic surface design, computational tools have assisted the development of materials for biomedical applications, oil–water separation, food packaging, water and energy harvesting, and biofabrication.^[^ ^182^ ^]^ There are several reviews in the literature that offer comprehensive information on the durability of superhydrophobic surfaces, challenges during commercialization, and low surface energy modifiers used in the development of superhydrophobic surfaces.^[^ ^183^, ^184^, ^185^, ^186^, ^187^, ^188^, ^189^ ^]^ There are also reviews regarding the use of colloidal particles from natural resources for superhydrophobic surface development by using agricultural waste, industrial byproducts, and natural materials.^[^ ^182^, ^190^, ^191^ ^]^ Although some of the reviews mention the importance of using computational tools for future design studies, their use still needs to be further implemented in superhydrophobic and superoleophobic surface design.^[^ ^14^, ^36^ ^]^

For control of the surface hydrophobicity, DFT has been used to calculate the contact angle,^[^ ^192^ ^]^ to understand water–surface interactions with polymer coatings,^[^ ^193^, ^194^ ^]^ and to show the reversible wettability of polymer (sodium bis(2‐ethylhexyl) sulfosuccinate‐doped polypyrrole, PPy‐AOT) for oil–water separation, together with the switchable wettability of the polymer membrane.^[^ ^193^ ^]^ In another study, researchers show that an increase in the C/O ratio in graphene causes an increase in the distance between water molecules and graphene oxide which explains the hydrophobicity due to the higher C/O ratio.^[^ ^194^ ^]^

DFT can also help the researchers to reveal mechanisms behind the hydrophobicity,^[^ ^195^, ^196^ ^]^ and to understand the effect of surface hydrophobicity on surface catalytic reaction efficiency.^[^ ^197^ ^]^

When larger models are needed, MD enables an understanding of the self‐assembly behavior of molecules and allows visualization of water spread over different surfaces with respect to time. This provides information on surface–water interactions leading to a deeper understanding of adhesion and contact angle measurements.^[^ ^135^, ^174^, ^176^, ^178^, ^179^, ^180^, ^198^, ^199^, ^200^, ^201^, ^202^, ^203^ ^]^

**Figure 11:**
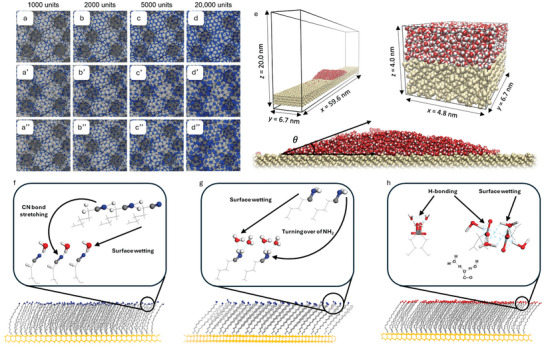
a–d″) Snapshots of water collected surfaces with respect to simulation time. a–d) Hydrophobic surface decorated with strong hydrophilic groups. (a′–d′) Superhydrophobic surface decorated with superhydrophobic group. (a″–d″) Superhydrophobic surface decorated with moderately hydrophilic groups. Reproduced with permission.^[^ ^178^ ^]^ Copyright 2020, American Chemical Society. e) Snapshots of water on native cellulose. Reproduced with permission.^[^ ^204^ ^]^ Copyright 2023, The Author(s). f–h) Behavior of functional groups of SAMs in their interaction with water. Functional groups of SAM (f) ─CN, (g) ─NH_2_, and (h) ─COOH. Reproduced with permission.^[^ ^199^ ^]^ Copyright 2011, American Chemical Society.

MD simulations also supported the development of a water harvesting material inspired by the hydrophilic structure of desert beetles for water drop nucleation and the hydrophobic structure of lotus leaves for water removal and transport. The simulation snapshots with respect to time are shown in **Figure** **11**a–d.^[^ ^178^ ^]^ The use of FA‐MD in this study enables the observation of water molecules over time at an atomistic level, which is not possible by using experimental methods.

A different study shows the formation of plant barrier layers and self‐assembly processes in cutin, providing inspiration to design new polymeric films for packaging and other applications with hydrophobicity, biodegradability, and low toxicity.^[^ ^174^ ^]^ MD simulations show that the secondary hydroxyl groups of the fatty acids in the plant‐barrier layer are responsible for 2D growth, whereas fatty acids with primary hydroxyl groups cause multilayer coverage. In a larger scale system, the self‐assembly and disassembly behavior of three biomimetic peptides under neutral and acidic conditions for drug delivery applications was studied using CG‐MD.^[^ ^202^ ^]^ This approach makes the research process of peptide design for target applications shorter than the time‐consuming experimental methods. The interaction of cellulose and acetylated cellulose with liquids of different polarities was studied using FA‐MD. The study provides contact angle and surface tension calculations. The cellulose‐liquid system and contact angle measurement are shown in Figure 11e. With this study, it is concluded that the acetylation of cellulose does not cause a higher affinity for nonpolar solvents.^[^ ^204^ ^]^ Combining DFT and MD, another study investigated the interaction of functionalized SAMs with water to develop superhydrophilic layers. When interacted with water, a ─CN end group shows stretching because of the N─H bond (Figure 11f), the ─NH_2_ group configuration turned over because of the H bonds between N and O from H_2_O (Figure 11g), and the ─COOH group shows a significant change in its structure because of the disruption of the dimer ─COOH structure by water (Figure 11h). To understand the surface wetting during the simulation, solvent‐accessible surface area was calculated. DFT calculations were used to calculate the binding energy of water molecules with the head groups.^[^ ^199^ ^]^ With MD and DFT, researchers could observe the direct interaction between water and functional groups at an atomic level, which helps develop surfaces with tailored wetting properties. Another study using FA‐MD simulations investigated the interactions of cellulose esters (cellulose acetate and cellulose acetate propionate) and water.^[^ ^205^ ^]^ The study reveals information beyond the reach of experimental methods. Understanding polymer–water interaction at the nanoscale might help to perform a bottom‐up design of hydrophobic surfaces FA‐MD also enables the study of the self‐assembly behavior and material properties of peptide sequences.^[^ ^200^ ^]^

To produce a superhydrophobic surface with antibacterial properties, a gecko‐inspired topology on a stainless steel surface was created by laser etching, functionalized with PDA (adhesive protein secreted by marine mussels), and grafted with octadecyl amine. The water adsorption behavior of the bare stainless steel and functionalized stainless steel was also shown by FA‐MD simulations.^[^ ^179^ ^]^ In another study, a silica coated varnished surface with a roughness property mimicking the coral‐reef structure was developed FA‐MD results provided information regarding the water concentration through a film. System energy calculations helped to understand water absorption on coatings.^[^ ^180^ ^]^

**Figure 12:**
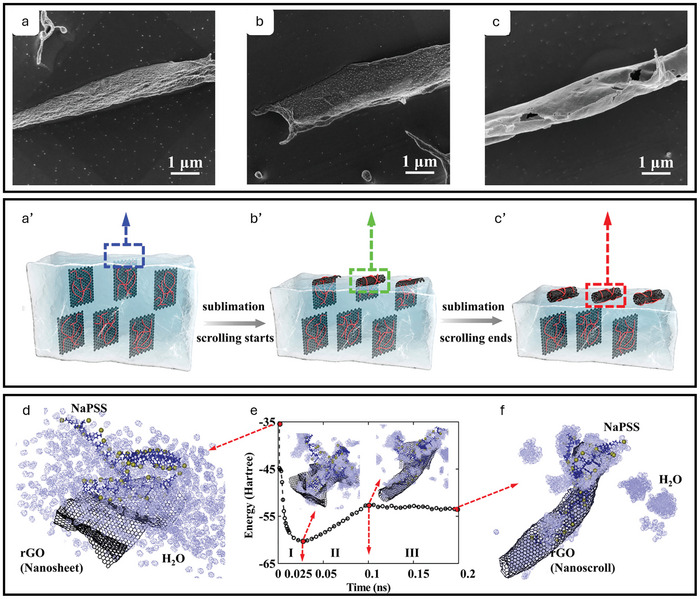
a) Cryo‐SEM the initial stage of the nanosheets. b) Cryo‐SEM, nanoscroll formation. c) Cryo‐SEM, nanoscroll formation completed by overlapping of the edges. a′) FA‐MD material in nanosheet forming at the fast freezing stage. b′) FA‐MD, nanosheet scrolling with the effect of ice sublimation. c′) FA‐MD nanoscroll formation completed after freeing from the ice of the material. d) FA‐MD initial stage of the system with planar material. e) Energy of the system with respect to time. Three stages are described with FA‐MD images which are I) dissolution, II) scrolling, and III) relaxation. f) FA‐MD, the final stage of the material in nanoscroll form. Reproduced with permission.^[^ ^198^ ^]^ Copyright 2018, American Chemical Society.

Another MD simulation study investigated how catechol‐containing comonomers affected the adhesion and mechanical properties of copolymer poly(*n*‐butyl acrylate‐*co*‐acrylic acid). The simulations provided insight into the cohesive‐adhesive properties of the polymers, their intramolecular, electrostatic, and van der Waals interactions, and the steric hindrances, which are not possible to observe by experimental methods. This informs further development of bioadhesives with tailored adhesive properties.^[^ ^201^ ^]^ For NO_2_ sensor development, researchers were inspired by a dog's sensitive sense of smell, and they mimicked the capillary structure inside a dog's nose using functionalized reduced graphene oxide sheets and a lyophilization technique. The team used FA‐MD to understand the nanoscroll formation from nanosheets and performed experiments with cryo‐SEM to validate their mechanism. The figures for cryo‐SEM images and related stages of the FA‐MD simulation are shown in **Figure** **12**a–c,a**′**–c**′**. In Figure 12d, the initial state of the simulation is given. In Figure 12e, the different stages of the simulation are shown based on the changes in the energy of the system, while the final stage is shown in Figure 12f.^[^ ^198^ ^]^ In this study, FA‐MD revealed the molecular scale mechanism of a nanoscroll formation, which is complementary to experimental data.

**Figure 13:**
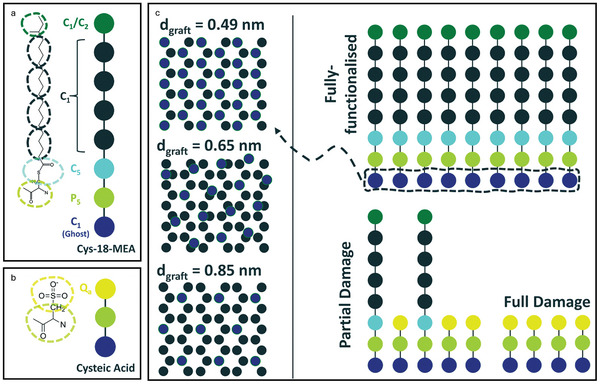
a) CG‐MD model of the Cys‐18‐MEA. 18‐MEA molecule represented by P_5_, C_5_, C_1_, and C_2_ beads. b) CG‐MD model of cysteic acid represented by C_1_, P_5_, and Q_a_ beads. Q_a_ bead represents the sulphonate group. c) Use of Cys‐18‐MEA and cysteic acid CG‐MD model structure to represent fully functionalized, partially damaged and fully damaged hair surface with different *d*_graft_ values. Reproduced with permission.^[^ ^176^ ^]^ Copyright 2022, The Royal Society of Chemistry.

CG‐MD simulations using the Martini force field were performed by researchers to investigate the surface properties and wetting behavior of hair with different damage ratios, mimicking the effect of bleaching on hair. They studied how changes in fatty acid molecules affect hair surface properties in various environments, including water and n‐hexadecane, and measured contact angles to validate their findings. The CG‐MD model for the study can be seen in **Figure** **13**a–c.^[^ ^176^ ^]^ They determined that fully functional hair is hydrophobic and oleophilic, but as damage increases, hydrophilicity increases, consistent with previous experiments, and can be modeled using the Cassie–Baxter wetting model.^[^ ^176^ ^]^ The detailed analysis of the hair surface with and without damage opened new avenues to control hair surface hydrophobicity in new cosmetic products.

AI can be used to overcome the otherwise high demands of experimental and numerical methods like FEM, allowing for many more design options to be identified and evaluated, the most promising of which can then be explored in experimental material development. By using ML, the studies target predicting contact angle values of the surfaces based on a pillar structure.^[^ ^199^, ^200^ ^]^ It is also possible to learn the most effective parameters that can determine the contact angle through ML.^[^ ^147^ ^]^ Another study also used ML for the prediction of SA on surfaces endowed with random pits.^[^ ^206^ ^]^

A method for the prediction of the wettability of the surfaces using ML was developed by researchers. In their study, they used SEM images of surfaces with different roughness's for the training of the ML algorithm. The model could also suggest optimum surface structures for superhydrophobicity and predict the wettability of new surface structures not used in input data during modeling.^[^ ^207^ ^]^ Another study also used ML for superhydrophobic surface design with different topographies. They benchmarked Artificial Neural Network, polynomial, support vector machine, Gaussian process regression, and Decision Tree regression. By combining experimental and numerical data with ML, they could reach a high design area for superhydrophobic material development.^[^ ^208^ ^]^

## 5 Conclusion

The development of superhydrophobic, underwater superoleophobic, and superamphiphobic surfaces has led to environmental concerns due to the accumulation of fluorinated compounds. However, research is underway to create sustainable alternatives inspired by Nature. While successful methods such as using nanotechnology, 3D printing, and templating exist for superhydrophobicity and underwater superoleophobicity, limited success has been achieved in creating sustainable in‐air superoleophobic surfaces. To overcome the challenges associated with in‐air superoleophobicity, biomimicry holds great promise. Researchers can learn from nature‐based strategies employed by organisms such as Leafhoppers and Collembola to develop more effective solutions. In addition to experimental developments, computational studies and tools play a crucial role in understanding the structure–property relationships of these materials. Computational methods like DFT, MD, and FEM can provide valuable insights into atomistic‐level phenomena. The integration of these computational methods with AI techniques, such as ML, offers exciting opportunities for optimizing and predicting material structures.

This review has showcased the recent advancements of superhydrophobic and superoleophobic surfaces, aided by bioinspiration. A summary of the bioinspired materials is shown in **Table** **2**. Bioinspiration, particularly from the Springtail, is providing a route to durable superoleophobicity without the use of fluorocarbon functionalities.

**Table 2:** A comparative summary of the key properties of the bioinspired materials.

Inspiration	WCA [°]	WSA [°]	OCA [°]	OSA [°]	Fluorocarbon functionality	Durability	Refs.
Rove beetles and water striders	160	9.8	N/A	N/A	No	Thermostable pH stable UV stable Abrasion resistant	[110]
Conch shells	154.4	3.8	N/A	N/A	No	Abrasion resistant Ductile	[112]
Diving bell spiders	146	N/A	N/A	N/A	No	N/A	[113]
Honeycomb	>150	N/A	Oleophilic	N/A	No	Thermostable Ductile Flame‐retardant Stable in water and seawater pH stability	[115]
Marine mussels	151.9	11.3	N/A	N/A	No	Washable Vulnerable to abrasion	[116]
Lotus leaf	164	2.6	Oleophilic	N/A	No	N/A	[111]
Lotus leaf	≈160	<5	N/A	N/A	No	N/A	[117]
Lotus leaf	153.9	8	N/A	N/A	No	pH stable UV stable O_2_ plasma stable Flame‐retardant	[118]
Goose feathers	153	N/A	N/A	N/A	No	N/A	[119]
Leather	161.5	N/A	152.2	N/A	Yes	Thermostable UV stable	[126]
Fish skin	<5	N/A	160–170 (underwater)	N/A	No	Oil/water separation efficiency unaffected by reuse; slight decrease in flux	[127]
Nacre	Hydrophilic	N/A	160 (underwater)	N/A	No	pH stable Solvent resistance Pressure stable High Young's modulus and hardness	[128]
Manis pentadactyla	N/A	N/A	≈115	N/A	No	Impact resistant	[130]
Springtail	156	N/A	130	N/A	Yes	Ductile Pressure stable Elastic	[122]
Springtail	>150	≈15	>150	≈10	Yes	Abrasion resistant Lifespan >6 months	[123]
Springtail	154.7	0.2	>150	<4	Yes	Pressure stable Elastic	[124]
Springtail	>150	≈2	>140	<10	Yes	Thermostable pH stable Ultrasonic cleaning resistant Scratch resistant Lifespan >120 days	[125]
Springtail	161.5	N/A	>150	N/A	No	O_2_ plasma stable Ductile Elastic Impact resistant	[133]
Springtail	158.6	N/A	>150	N/A	No	O_2_ plasma stable Ductile Elastic Impact resistant	[134]

Furthermore, computational tools can successfully predict the wetting properties of surfaces and provide atomistic‐level information with respect to time, which is not possible with experimental methods. These tools can be used to predict the contact angle of the surfaces for different liquids in the experimental range and to observe the liquid behavior on the surfaces, such as diffusion or adsorption. They can also virtually observe changes in inter‐ and intramolecular interactions, such as hydrogen bonds, throughout the simulation, which affect the observable experimental properties of the materials, such as form, stiffness, and water‐related properties. Computational tools allow us to follow specific atoms’ behavior over time, and this provides information regarding functional groups interacting with water, while identifying the forces governing this interaction. Understanding the governing interactions gives researchers the opportunity to control the synthesis processes and to make more accurate designs for their experiments. Additionally, AI and ML methods are accelerating material design.

### 5.1 Future Directions and Outlook

Future directions should be based on successful methods thus far, whilst investigating alternatives to the energy extensive processes. Up to now the only avenue to produce the precise, nanoscale re‐entrant structures required for effective superoleophobic surfaces without incorporating fluorocarbons involves the use of photolithography. In the cases seen, this is used with a templating method which somewhat reduces the energy intensity of the process, as the molds created through photolithography can be reused many times. However, even the templating technique is costly, energy intensive, and usually uses harmful chemicals for etching. More systematic studies of the direct effect of dimension sizes on oleophobicity to ascertain the essential requirements are required. When the specifications are known, other potential methods of production can be identified and investigated.

3D printing is promising especially as technological advancements allow for easier creation of smaller feature sizes. Nevertheless, it is still expensive and time‐consuming to produce large volumes, particularly when a combination of techniques is required. This is the case for potential superoleophobic materials. Macroscale printing is used for the main substrate of the material, while micro‐ and nanoscale printing are used to produce hierarchical, re‐entrant structures. 3D printing could be implemented to print molds, as discussed with photolithography. The molds could then be used repeatedly to produce the final structured materials, if demolding is achievable, thus reducing cost and time per output made, and making the method more feasible for industrial scale.

Nanosecond lasering is another prospective method as it can be used to produce nanoscale features on a range of substrates including metals, plastics, glass, and ceramics. Subsequent steps still need to be evaluated to validated nanosecond lasering on biobased polymer materials. The features produced with this technique are less precisely tuneable, but consistent re‐entrant structures across a surface have been achieved, as discussed. Compared to femtosecond lasers, nanosecond lasers are more cost‐effective with simpler technology, the processing time is faster and so they are more suitable for large scale production. Femtosecond lasering is also a potential option, with higher precision for nanostructures but is more costly and time consuming per unit of material produced. Again, evaluations into the compulsory feature dimensions would provide further insights, if the intensity of femtosecond lasers is required, or if nanosecond lasers would be sufficient.

Shrinking techniques such as thermoresponsive hydrogels or pyrolysis methods are being used to reduce the dimensions of features. These reduce the strenuousness of the initial fabrication method, as the size restrictions are not so stringent. As seen in this review, shrinking was only applied to structures made through photolithography. Therefore, it would be formative to research this further with features made by other processes and determine if the shrinking effect can be applied to a more cost and energy efficient initial method. For example, with lasering techniques, using a shrinking technique could allow for femtosecond lasering‐like structures to be produced through nanosecond lasering and shrinking, reducing the energy intensity of the process whilst maintaining the final result.

Electrospinning increases the roughness of a material, which enhances its inherent wettability properties. This is pertinent in the case of a hydrophobic polymer, which can often become superhydrophobic after electrospinning, but this method has not been able to induce superoleophobicity. This is likely because the material needs to already have oleophobicity before electrospinning, meaning the initial property comes from chemistry rather than structure, and so it is difficult to achieve superoleophobicity without the use of fluorocarbons. Furthermore, post processing is required to produce a usable film or coating from the electrospun polymer, although this can reduce the roughness and so the liquid repellency of the material. Despite this, if a superrepellent film or coating is successfully fabricated, it could then be applied to an independent substrate. Then, a sustainable material could be chosen such as cardboard, and the repellent properties of the film or coating would be donated to the cardboard, although this again mandates another fabrication step of applying the coating to its substrate, which would also require research into the most effective, efficient, and scalable application method.

SAMs allow exact control of the surface chemistry, with functional groups added relatively easily depending on chemical compatibility. The thickness of the layers is also directly controllable, and they form spontaneously so the process is energy efficient and feasibly scalable. As the layers are formed through chemical bonding, they are usually stable and durable. The method is already used industrially in microelectronics, biosensors, and more, and has proven sufficient for sustainable superhydrophobic materials. However, the repellency properties are mostly a consequence of surface chemistry, so the resultant materials may never be able to achieve superoleophobicity without the use of fluorocarbons as the topography is not explicitly controllable.

To further assist the development of the materials, computational tools need to be furthered integrated into the design process. Nevertheless, to fully harness the potential of computational tools, future studies should focus on combinations of simulation methods (DFT, MD, etc.) with ML algorithms. The role of experimental findings on the application of ML is also significant, because the training of ML models depends on the accuracy of the experimental data. Therefore, future studies need more collaboration between researchers with expertise in computational and experimental methods to gain better insight into surface design parameters.

Another point discussed for using computational tools in surface design is the test methods used for the analysis of the simulations. Although computer‐based methods give more control over test environments and conditions, differences in simulation parameters and methods for the analysis of simulation data, as seen in contact angle and diffusion analysis, make more challenging the comparability of computational results across studies. While fully standardizing the set‐up of the simulations is extremely challenging given the differences in systems, test conditions and computational method, some degree of standardization is needed to enable more accurate comparisons.

In summary, although the current simulation methods are quite helpful in understanding the atomistic level behavior of the surfaces and materials, they have still limitations in the length and time scale of the models and simulations. With the development of more advance high throughput, GPU‐enabled, and quantum computing technologies, and with the implementation of more accurate ML models for material discovery and property prediction, it will be possible to achieve larger models and longer simulations. These improvements will allow faster scanning of design options, which will contribute to a better understanding of governing parameters in surface design, reduce the time of computational‐to‐experimental data validation.

## Conflict of Interest

The authors declare no conflict of interest.
